# Statin-Induced Myositis with Concomitant Myocarditis

**DOI:** 10.7759/cureus.31871

**Published:** 2022-11-24

**Authors:** Haris Patail, Aditi Kothari, Vidya Nadig, Jordan Kunkes

**Affiliations:** 1 Internal Medicine, University of Connecticut Health, Farmington, USA; 2 Cardiology, Hartford Hospital, Hartford, USA

**Keywords:** cardiac mri, high-sensitivity troponin, creatine kinase, internal medicine, rheumatology, cardiology, hmg coa reductase, myocarditis, myositis, statin

## Abstract

Statins are commonly prescribed medications that provide many significant cardiovascular benefits for both primary and secondary prevention in patients with and without coronary artery disease. Known adverse effects of these medications include varying degrees of muscle toxicity, including myalgia, myopathy, and rare cases of necrotizing myositis, hepatic dysfunction, and central nervous system changes. Despite known adverse effects, statins are rarely associated with myocarditis. Statins can cause skeletal muscle myopathy and myositis by upregulating HMG-CoA reductase (HMGCR) in muscle tissue, resulting in antibody-mediated inflammation. A similar proposed mechanism is likely possible within cardiac myocytes. We present a rare case of statin-induced necrotizing myositis with concomitant cardiac involvement. Severe skeletal muscle myositis was confirmed by lower extremity MRI and biopsy findings. In association, elevated and plateaued high-sensitivity troponin without evidence of cardiac ischemia warranted cardiac MRI, which further confirmed myocarditis due to inflammation within a non-vascular distribution. Given its rare presentation, the treatment for statin-induced cardiac toxicity is unclear; however, the patient in this case report was treated with pulse-dose intravenous steroids and indefinite discontinuation of statin medications.

## Introduction

Statins provide many significant cardiovascular benefits and are generally reported as well-tolerated medications [1-6]. We present the case of an 80-year-old male who had been experiencing lower extremity weakness and fatigue for several months and was found to have inflammatory myositis and myocarditis. He had been taking atorvastatin 40 mg daily for nearly two years and was found to have positive HMG-CoA reductase (HMGCR) autoantibody, raising concern for statin-induced muscle and cardiac toxicity. Muscular toxicity is considered a result of the up-regulation of HMGCR in muscle tissue, resulting in antibody-mediated autoimmune necrosis [7]. Statin-induced myositis with associated myocardial involvement has only been reported in one previous study in the literature [8].

## Case presentation

An 80-year-old male with a medical history of type 2 non-insulin-dependent diabetes mellitus, hypertension, hyperlipidemia, diastolic heart failure, and atrial fibrillation presented to the hospital after three months of fatigue and progressive lower extremity weakness, specifically in the proximal muscles. Prior to these presentations, he was working full-time and independently completing his daily-life activities. He denied recent viral illness or sick contacts and confirmed compliance with chronic medications, including aspirin, atorvastatin, apixaban, losartan, metformin, and metoprolol. 

At the time of admission, his vitals were as follows: temperature of 95.5F, heart rate of 50 beats per minute, blood pressure of 110/50 mmHg, and oxygen saturation on room air of 98%. Physical exam was significant for 3/5 strength in the bilateral hip flexors and extensors, 5/5 strength in the distal lower extremity, and 5/5 strength in the upper extremities. Complete blood count and basic metabolic panel were within normal limits. Notable lab results included aspartate aminotransferase (AST) of 259 U/l, alanine aminotransferase (ALT) of 209 U/l, creatinine kinase (CK) of 4,418 U/l, aldolase of 41.8 U/l, high-sensitivity troponin T of 949 ng/l, C-reactive protein (CRP) of 1.8 mg/dl, and erythrocyte sedimentation rate (ESR) of 56 mm/hr. Electrocardiogram (EKG) showed atrial fibrillation with a slow ventricular response without ST-segment changes.

He was initiated on pulse-dose intravenous corticosteroids due to suspicion of myositis and was admitted to the medicine service for further workup. Antinuclear antibody (ANA) titer was weakly positive at 1:40, and rheumatoid factor (RF) was slightly elevated at 19 IU/ml. Double stranded-DNA (ds-DNA) antibodies, cyclic citrullinated peptide (CCP) antibodies, anti-Ro, anti-La, antineutrophil cytoplasmic antibodies (ANCA), proteinase 3 antibodies, and myeloperoxidase antibodies were negative. Urine toxicology, human immunodeficiency virus (HIV), rapid plasma reagin (RPR), and Lyme IgM/IgG were negative. Notably, the patient's HMGCR antibody was positive at 55, and the myositis autoantibody panel was negative. Magnetic resonance imaging (MRI) of the lower extremities showed increased T2 signaling in all muscles of the hip and thigh bilaterally, predominantly within the tensor fascia lata and vastus lateralis. This increased the suspicion of inflammation and edema consistent with non-infectious myositis. Subsequent right rectus femoris muscle biopsy showed active myofiber necrosis and phagocytosis, perivascular lymphocytic infiltration, and mild endomysial fibrosis without evidence of amyloid deposition or inclusion bodies, confirming statin-induced myositis.

**Figure 1:**
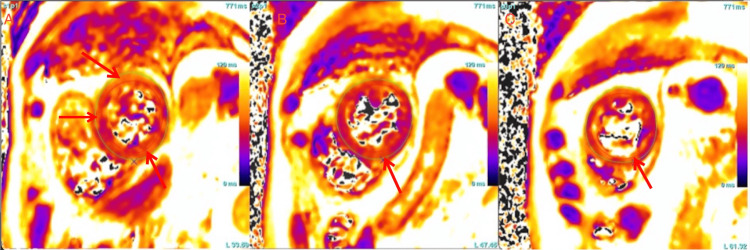
Cardiac MRI, short-axis T2 mapping from the cardiac base (A) to the apex (C) showing elevated T2 relaxation time within septal and lateral myocardial walls, consistent with edema and inflammation with a non-vascular distribution.

**Figure 2:**
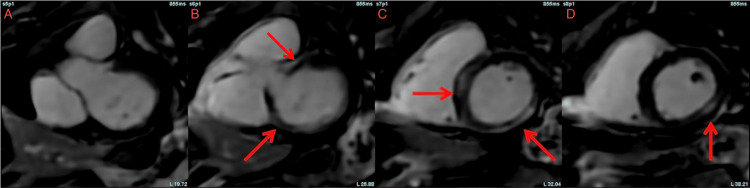
Cardiac MRI, short-axis stack of the left ventricle showing mid-myocardial (B) to epicardial (C and D) late gadolinium enhancement along the basilar lateral wall and basilar inferoseptal wall. The enhancement is consistent with T2 mapping for inflammation with a similar non-vascular distribution.

High-sensitivity troponin remained elevated throughout the hospitalization period and plateaued at over 900. Due to the suspicion of myocardial involvement, the patient underwent a transthoracic echocardiogram (TTE), which revealed a moderately dilated left atrium, normal left ventricular systolic function with an ejection fraction of 63%, and no wall motion abnormalities. This was relatively unchanged from prior echocardiograms. Since the patient had not reported chest pains nor the EKG findings indicated ischemia, a coronary angiogram was deferred because an acute coronary syndrome was ruled out. He ultimately underwent cardiac MRI (Figure 1 and Figure 2), which revealed patchy mid-myocardial to epicardial enhancement along the basilar septum and inferoseptal base. This enhancement was noted to be non-vascular and non-ischemic in distribution, indicating myocarditis.

## Discussion

HMGCR inhibitors, also known as statins, are lipid-lowering medications used to treat hypercholesterolemia and prevent cardiovascular events as both primary and secondary prevention [1-3]. Patients with known coronary artery disease have been shown to benefit from statin therapy through the improvement of cardiovascular disease (CVD) in the setting of ischemic heart disease [4,5]. For secondary prevention, the United States Preventive Services Task Force (USPSTF) recommends statins for patients between the ages of 40 and 75 without known coronary heart disease but with increased CVD risk due to comorbidities such as hyperlipidemia, diabetes, hypertension, or smoking, and with a calculated atherosclerotic cardiovascular disease risk (10-year CVD risk) greater than 10% [1].

As statins are commonly used worldwide, they have generally been reported as well-tolerated medications [3,6]. Statin-associated muscle symptoms and myalgia are the most commonly reported adverse effects, affecting up to 10% of patients [6]. Statin-induced necrotizing myositis (SINM) is a rare but serious complication that leads to proximal muscle weakness, significantly elevated creatinine kinase (CK) levels, and detected antibodies against HMGCR [6]. The proposed mechanism of SINM involves the up-regulation of HMGCR within muscle tissue, which becomes a target for autoantibodies for inducing autoimmune necrosis [7]. SINM can occur in patients who have been receiving long-term statin therapy [9]. A large systematic review found that patients with a mean age of 65 who had been on statins for over three years subsequently developed inflammatory myositis [10]. 

Statin-induced myositis with concomitant myocarditis has only been reported once in prior research [8]. This previous case concluded that, like the mechanism of myocarditis is secondary to statin therapy, SINM is secondary to the upregulation of HMGCR in muscle tissue. It is commonly known that statins may be used as an adjunctive treatment for the management of myocarditis by modifying oxidation, inflammation, immunomodulation, and endothelin activity [11,12].

## Conclusions

While statins remain integral to both primary and secondary prevention of adverse cardiac events, they are not without risk. Though rare, complications more severe than myalgias and transaminitis may occur in statin therapy. We conclude that our patient’s unfortunate case of necrotizing myositis and myocarditis was induced by statin use. Supporting evidence includes his progressive proximal muscle weakness, elevated CK and aldolase, positive HMGCR antibody, active myofiber necrosis on muscle biopsy, elevated high sensitivity troponin without ischemic evidence on EKG or TTE, and non-ischemic myocardial enhancement on cardiac MRI suggestive of myocarditis. Given the absence of literature, studies should be directed toward establishing a plausible correlation between statin use and acute myositis complicated by myocarditis.
